# Supplementary material for: Caffeine and Chlorogenic Acid Combination Attenuate Early-Stage Chemically Induced Colon Carcinogenesis in Mice: Involvement of oncomiR miR-21a-5p
Source: Int J Mol Sci. 2022 Jun 4;23(11):6292. doi: 10.3390/ijms23116292 (PMC9181067; doi:10.3390/ijms23116292)
Supplement: Supplementary file 1 [file ijms-23-06292-s001.zip › ijms-1727504-supplementary.pdf]

Table S1. Experimentally validated targets of mmu-miR-21a-5p.

Target gene	Database	Confidence
Fasl	miRTarbase, miRDB	Reporter assay and Western blot
Peli1	miRTarbase, miRDB, TargetScan	qPCR and Western blot
Pdcd4	miRTarbase, miRDB, TargetScan	Reporter assay, pPCR and Western blot
Spry2	miRTarbase, miRDB, TargetScan	Reporter assay and Western blot
Pten	miRTarbase	Reporter assay, pPCR and Western blot
Reck	miRTarbase, miRDB, TargetScan	Reporter assay, pPCR and Western blot
Spry1	miRTarbase, miRDB, TargetScan	Reporter assay and Western blot
Tgfbi	miRTarbase, miRDB, TargetScan	Reporter assay, pPCR and Western blot
Pdcd4	miRTarbase, miRDB, TargetScan	Others
Btg2	miRTarbase, miRDB, TargetScan	Others
Spry4	miRTarbase,	qPCR
Spry3	miRTarbase	Western blot
Elavl4	miRTarbase	Others
Pias3	miRTarbase	Western blot
Tgfbr3	miRTarbase	Reporter assay, pPCR and Western blot
Tnfaip8l2	miRTarbase	Reporter assay, pPCR and Western blot
Smad7	miRTarbase, miRDB, TargetScan	qPCR and Reporter assay
Gt(ROSA)26Sor	miRTarbase	qPCR and Reporter assay
Yy1	miRTarbase	qPCR and Reporter assay
Eif4e3	miRTarbase	qPCR and Reporter assay
Pdcd10	miRTarbase	qPCR and Reporter assay
Timp3	miRTarbase, miRDB, TargetScan	qPCR
YOD1	miRTarbase, miRDB, TargetScan	Reporter assay, pPCR and Western blot
PDCD4	miRTarbase, miRDB, TargetScan	qPCR and Western blot
Mmp9	miRTarbase	qPCR and Western blot
Kcnk6	miRTarbase	NGS
Map3k1	miRTarbase, miRDB, TargetScan	NGS
Cyfip1	miRTarbase	NGS
Rmnd5a	miRTarbase, miRDB, TargetScan	NGS
Tns1	miRTarbase, TargetScan	NGS
Gid4	miRTarbase, miRDB, TargetScan	NGS
E2f2	miRTarbase	NGS
Rpp40	miRTarbase	NGS
Moap1	miRTarbase	NGS
AK010878	miRTarbase	NGS
